# A262 NEONATAL ACUTE LIVER FAILURE DUE TO PRESUMED GESTATIONAL ALLOIMMUNE LIVER DISEASE - A CASE REPORT

**DOI:** 10.1093/jcag/gwac036.262

**Published:** 2023-03-07

**Authors:** M Schmidt, S Ling, V Ng, B Kamath, S Kortbeek, N Jones, M Miserachs, N Lepore, N Reitzel, M Zachos, K Prowse, B Syed, A Sidhu, S Shurrab, M Kozenko, R Bandsma

**Affiliations:** 1 Gastroenterology, Hepatology and Nutrition, The Hospital for Sick children, Toronto; 2 Pediatric Gastroenterology, Nutrition and Hepatology, McMaster Children's Hospital, Hamilton; 3 General Surgery, The Hospital for Sick children, Toronto; 4 Neonatology; 5 Pediatrics, McMaster Children's Hospital, Hamilton, Canada

## Abstract

**Background:**

Neonatal acute liver failure (NALF) is a rare disease that is distinct from acute liver failure seen in older children and adults. Gestational alloimmune liver disease (GALD) is the most frequent cause, is initiated in utero by sensitization of the maternal immune system to a fetal hepatocyte antigen and subsequent production of maternal immunoglobulin G antibodies that cross the placenta. Maternal IgG binds to a fetal hepatocyte antigen and initiates an innate immune response involving the terminal complement cascade and membrane attach complex. The understanding of the alloimmune origin has led to the use of intravenous immunoglobulin (IVIG) treatment and exchange transfusion, significantly increasing survival. However, approximately 25% of patients may not respond and require salvage liver transplantation. In spite of an increased rate of comorbidities, concern for technical difficulties and limited graft availability, young infants eligible for transplant have been shown to have similar overall patient and graft survival rates compared to older children with other indications for liver transplant.

**Purpose:**

The primary aim of our study is to report a case of NALF with successful liver transplant.

**Method:**

We present the case of a preterm girl with NALF due to GALD refractory to medical management, requiring liver transplantation.

**Result(s):**

This is a 35-week preterm girl, with scant pre-natal care, birth weight of 1.825 kg and Apgar 9/9. She is the seventh child of non-consanguineous parents, with healthy siblings. On day-of-life (DOL) 1 she presented with acute kidney injury, progressive worsening metabolic acidosis and hyperammonemia and was found to be profoundly coagulopathic (INR 6), with normal liver enzymes and liver failure was diagnosed. Initial investigation ruled out congenital infections, sepsis, neonatal hemophagocytic lymphohistiocytosis and metabolic diseases. Magnetic resonance imaging of the body demonstrated findings in keeping with iron deposition in the thyroid, liver and pancreas, suggestive of GALD. Completed double volume exchange transfusion and IVIG on DOL 9 and repeat IVIG on DOL 13 and 15, with partial improvement in INR. Due to persistent ascites, conjugated hyperbilirubinemia and hyperammonemia she was transferred for urgent liver transplant assessment. Persistent liver dysfunction in the form of hyperammonemia, hypoglycemia and progressive coagulopathy led to transplant listing on DOL 30. ABO incompatible deceased donor liver transplant was completed on DOL 62 (4.075 kg, estimated dry weight 3.5 kg). The procedure was uncomplicated, liver enzymes normalized, coagulopathy and hypoglycemia resolved. She was transferred to the ward on post-operative day (POD) 6. and weaned off sedatives and transitioned to oral feeds within 2 weeks of transplant, with complex abdominal wound closure on POD 29.

**Conclusion(s):**

Successful liver transplantation is possible in neonates with acute liver failure due to GALD refractory to medical management and weighing 4kg or less.

**Please acknowledge all funding agencies by checking the applicable boxes below:**

None

**Disclosure of Interest:**

None Declared

MICROBIOME & MICROBIAL THERAPY

